# Total intravenous anaesthesia in a goat undergoing craniectomy

**DOI:** 10.1186/s12917-017-1205-2

**Published:** 2017-09-15

**Authors:** Verónica Vieitez, Ignacio Álvarez Gómez de Segura, Víctor López Rámis, Massimo Santella, Luis Javier Ezquerra

**Affiliations:** 1 Veterinary Teaching Hospital, University of Extremadura, Avda, Universidad s/n, 10003 Cáceres, Spain; 2 Department of Animal Medicine and Surgery, Veterinary Faculty, University Complutense of Madrid, Avda. Puerta de Hierro, 28040 Madrid, Spain

**Keywords:** Goat, Propofol, Fentanyl, Lidocaine, Midazolam, Craniectomy, Total intravenous anaesthesia

## Abstract

**Background:**

Cerebral coenurosis is a disease of the central nervous system in sheep and goats, and is usually fatal unless surgical relief is provided. Information regarding neuroanaesthesia in veterinary medicine in goats is scant.

**Case presentation:**

We describe anaesthetic management of an intact female goat (2 years; 16 kg) presented for craniectomy. The goat was sedated with xylazine (0.05 mg kg^−1^, i.m.) and morphine (0.05 mg kg^−1^, i.m.). General anaesthesia was induced 20 min later with propofol and maintained with a constant rate infusion of propofol (0.2 mg kg^−1^ min^−1^). A cuffed endotracheal tube was placed and connected to a rebreathing (circle) system and mechanical ventilation with 100% oxygen was initiated. A bolus of lidocaine (1 mg kg^−1^), midazolam (0.25 mg kg^−1^) and fentanyl 2.5 μg kg^−1^ was delivered via the intravenous route followed immediately by a constant rate infusion of lidocaine (50 μg kg^−1^ min^−1^), midazolam (0.15 mg kg^−1^ h^−1^) and fentanyl (6 μg kg^−1^ h^−1^) administered via the intravenous route throughout surgery. Craniectomy was undertaken and the goat recovered uneventfully.

**Conclusion:**

Total intravenous anaesthesia with propofol, lidocaine, fentanyl and midazolam could be an acceptable option for anaesthesia during intracranial surgery in goats.

## Background

Cerebral coenurosis is a disease of the central nervous system (CNS) in sheep and goats. Due to development of the cyst in brain, the animal starts showing nervous signs that include circling, visual defects, gait abnormalities, hyperesthesia, and paraplegia [1]. Surgical removal is the only available treatment. However, there are few reports of surgical treatment [1–4] and only brief descriptions of anaesthetic management. In those studies, goats were sedated [3] or controlled manually by an assistant [2] while surgery was undertaken under local anaesthesia. Only Merbl et al. [1] described two cases of constant rate infusion (CRI) of propofol and fentanyl.

Anaesthesia for intracranial neurosurgical procedures must provide haemodynamic stability, maintain cerebral perfusion pressure (CPP), reduce the cerebral metabolic rate (CMR), preserve cerebral autoregulation and avoid an increase in intracranial pressure (ICP) [5]. Anaesthesiologists must plan for smooth emergence, rapid awakening, and early neurological assessment of the patient in the postoperative period [6].

Volatile and intravenous anaesthetic agents are used for the maintenance of anaesthesia during neurosurgical procedures. Which type of anaesthetic (inhalational or intravenous) is preferable for a craniectomy is controversial. Volatile anaesthetics are dose-dependent cerebral vasodilators that affect cerebral autoregulation and ICP [7]. However, most intravenous anaesthetic, analgesic and sedative agents maintain a linear relationship between a reduction in cerebral blood flow (CBF) and CMR, autoregulation of cerebral perfusion and CO_2_ responsiveness, and have minimal effects on ICP [7].

In human medicine propofol does not interfere with CBF regulation [8] and decreases CMR, which stimulates vasoconstriction and results in decreased ICP in rats [9]. Clinical studies have suggested that propofol alone or in combination with alfentanil can aid the maintenance of anaesthesia during craniotomy in human beings [10].

Pure mu-agonist opioids (e.g. fentanyl) are commonly used for analgesia in balanced total intravenous anaesthesia (TIVA) and can attenuate nociceptive signals from noxious stimuli at subcortical levels of the CNS [11]. In goats, fentanyl has been shown to be effective against thermal and mechanical stimuli using a model of nociception [12].

Benzodiazepines such as midazolam contribute to TIVA through sedation and muscle relaxation [13]. Midazolam has been reported to be a potent sedative drug [14] that can be administered during the pre-anaesthetic period to reduce the dose of propofol required for induction of general anaesthesia in goats [15].

Intravenous lidocaine has been used as a supplement to general anaesthesia, and its perioperative administration reduces postoperative pain. Intravenous lidocaine has been shown to reduce the minimum alveolar concentration of isoflurane in dogs [16], cats [17], horses [18] and goats [19].

There is limited information regarding neuroanaesthesia in veterinary medicine. Moreover, goats are anaesthetised rarely, so information on the efficacy of anaesthetic drugs in this species is scant [13]. Maintenance of anaesthesia using propofol administered by CRI in combination with other injectable drugs or inhalation agents has been reported in goats [19–21]. Fentanyl, midazolam [21] and ketamine [20] have been shown to be anaesthetic-sparing agents that lower the propofol dose required for the maintenance of anaesthesia.

Here, we describe anaesthetic management in a goat undergoing resection of an intracranial coenurosis. Concurrent use of propofol, fentanyl, lidocaine and midazolam in goats has not been reported.

## Case description

An intact female goat (2 years old; 16 kg) was admitted to hospital for investigation of neurological signs. Behavioural disturbances, standing apart from other goats, and disorientation were observed. Furthermore, it seemed to be very easy to chase and catch the goat.

**Fig. 1:**
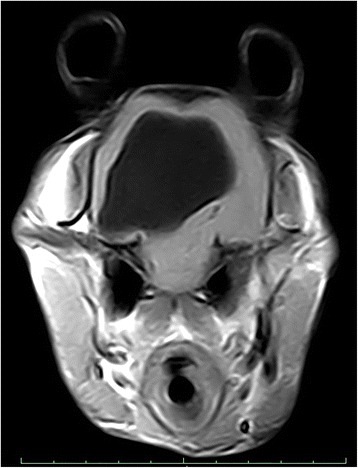
Axial T1 magnetic resonance image showing a hypointense lesion occupying most of the right hemisphere suspected to be a cerebral cyst

General physical examination was unremarkable. However, upon neurological examination, an absent left-sided menace response, alert mentation and head tilt were noted. No other abnormalities were noted and the results of haematology and blood biochemistry were unremarkable. Magnetic resonance imaging (MRI; Vet-MR Grande; Esaote S.A., Barcelona, Spain) of the brain revealed a cystic lesion at the level of the right cerebral hemisphere (Fig. 1). The goat was scheduled for resection of this lesion.

The goat was anaesthetised for surgery. Food (but not water) was withheld 12 h before anaesthesia. The goat was pre-medicated with xylazine (0.05 mg kg^−1^, i.m.; Rompun 2% soluble injection; Bayer, Barcelona, Spain) and morphine (0.05 mg kg^−1^, i.m.; Morfina 1%; Fresenius Kabi España S.A., Barcelona, Spain) 20 min before the induction of anaesthesia. A 20-G catheter (BD Insyte; Becton Dickinson Medicals Pte. Ltd., Singapore) was inserted into the cephalic vein for the administration of drugs and intravenous fluids. An antibiotic (ampicillin, 20 mg kg^−1^, i.v.; Britapen, 500-mg soluble injection; Reig Jofre S.A., Madrid, Spain) was given and administration repeated every 90 min throughout the surgical procedure. The auricular artery in the left ear was catheterised using a 24-G catheter. Five minutes before the induction of anaesthesia, the goat underwent pre-oxygenation via a facemask with 100% oxygen. Before the induction of anaesthesia, the degree of sedation was moderate. Propofol (Vetofol, 10 mg mL^−1^; Esteve, Barcelona, Spain) was administered initially as a bolus at 2 mg kg^−1^ over 30 s, followed by incremental doses at 0.5 mg kg^−1^ every 15 s, until the goat was anaesthetised sufficiently to allow endotracheal intubation (total dose, 50 mg). A cuffed, 6.5-mm ID endotracheal tube was placed and connected to a rebreathing (circle) system. Intermittent positive pressure ventilation (IPPV) with 100% oxygen was initiated using a pressure-cycled mechanical ventilator (ABC; Stephan GmbH, Gackenbach, Germany). Peak inspiratory pressure (PIP) was set at 8–10 cmH_2_O, and the respiratory rate (*f*_R_) adjusted to maintain a partial pressure of expired carbon dioxide (*P*E′CO_2_) at 33–35 mmHg.

The goat was placed in sternal recumbency for preparation of the surgical site with the head elevated at 30°. Occlusion of the jugular vein was avoided throughout the procedure. The goat was connected to a multi-parameter anaesthesia monitor (S/5; Datex-Ohmeda, Inc., Madison, WI, USA), which displayed continuously a lead-II electrocardiogram, heart rate (HR), *f*_R_, *P*E′CO_2_, inspired fraction of oxygen, tidal volume, invasive blood pressure (IBP), PIP, dynamic respiratory system compliance and plethysmographic oxygen saturation (SpO_2_). The catheter in the auricular artery was connected to an electronic pressure transducer (Deltran DPT-200; Utah Medical Products, Inc., Midvale, UT, USA) zeroed at the level of shoulder joint to measure IBP. Body temperature was measured using an intra-oesophageal temperature probe and displayed continuously on the multi-parameter monitor.

A CRI of propofol (6 mg kg^−1^ h^−1^) was started immediately after the induction of anaesthesia. Additionally, a bolus of lidocaine (1 mg kg^−1^; Lidocaína 2% soluble injection; B. Braun Medical S.A., Barcelona, Spain), midazolam (0.25 mg kg^−1^; Dormicum 5 mg mL^−1^ soluble injection; Roche Farma S.A., Madrid, Spain) and fentanyl (2.5 μg kg^−1^, Fentanest 0.05 mg mL^−1^ soluble injection; Kern Pharma, Barcelona, Spain) were delivered (i.v.) by hand over 60 s, followed immediately by a CRI of lidocaine (50 μg kg^−1^ min^−1^), midazolam (0.15 mg kg^−1^ h^−1^) and fentanyl (6 μg kg^−1^ h^−1^) administered via the intravenous route throughout the surgical procedure. Propofol and fentanyl for CRI were drawn up to fill a 20-mL syringe (Perfusor Compact; B. Braun Medical S.A.), whereas 600 mg of lidocaine and 30 mg of midazolam were mixed in 1 L of lactated Ringer’s solution (Ringer Lactato; B. Braun Medical S.A.) and infused together with a volumetric pump (Niki: C.M.E. Ltd., Lichtenstein, Germany) at 5 mL kg^−1^ h^−1^.

The criteria used to assess the depth of anaesthesia were HR, IBP, attempts to breathe against the ventilator and spontaneous movement. According to clinical signs, CRI of propofol was increased to 12 mg kg^−1^ h^−1^ 10 min after the induction of anaesthesia. Lactated Ringer’s solution was infused via the intravenous route during surgery at 5 mL kg^−1^ h^−1^. A bolus of propofol (0.5 mg kg^−1^, i.v.) was administered if voluntary movement occurred. The infusion rate of propofol was recorded every 5 min, along with all physiological parameters displayed on the multi-parametric monitor.

Samples of arterial blood were collected anaerobically from the arterial catheter. They were analysed immediately and 45 min after the induction of anaesthesia to ensure blood gases remained within established reference ranges (*P*aCO_2_, 39.5 mmHg; *P*aO_2_, 475 mmHg; HCO_3_, 29.5 mmol L^−1^; base excess, 5 mmol L^−1^; SaO_2,_ 100%; pH 7.481; lactate, 0.88 mmol L^−1^).

**Fig. 2:**
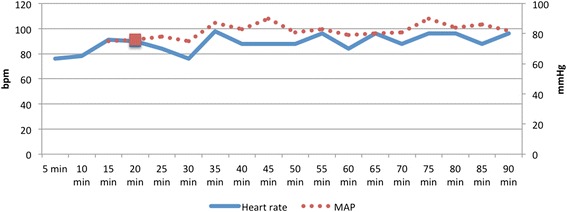
Heart rate (bpm) and mean arterial blood pressure (mmHg) in a goat undergoing craniectomy anaesthetized with TIVA of propofol, lidocaine, fentanyl and midazolam. Square marks the beginning of surgery

TIVA provided adequate cardiovascular stability (HR, 76–96 bpm) and adequate arterial blood pressure (Fig. 2) throughout anaesthesia (mean arterial blood pressure (MAP), 75–90 mmHg). Regurgitation, hyper-salivation and tympany were not observed. Oesophageal temperature decreased gradually over the period of anaesthesia but hypothermia (36.1 °C) was not corrected until the end of the procedure.

Craniectomy was undertaken to remove the cyst. Five minutes before completion of surgery, a bolus of dexamethasone (0.3 mg kg^−1^, i.v.; Cortesona 2 mg mL^−1^ soluble injection; Laboratorios Syva, León, Spain) and acepromazine (0.02 mg, i.v.; Equipromacina 5 mg mL^−1^ soluble injection; Labiana Life Sciences, S.A., Barcelona, Spain) were administered to ensure a smooth recovery. After skin closure, all CRI was stopped (total dose of propofol, 310 mg). IPPV was continued until attempts were made to breathe spontaneously. Then, the goat was moved in sternal recumbency to the recovery room. To avoid complications associated with the digestive tract and respiratory system, the goat was supported in sternal recumbency and the endotracheal tube removed only after return of the swallowing reflex (20 min after the end of the surgery). Total anaesthesia time (from the induction of anaesthesia to TIVA cessation) was 90 min. The goat recovered uneventfully from anaesthesia and could stand and eat <24 h after surgery.

Assessment of pain and physiological variables was conducted every hour until hospital discharge: no abnormalities were detected. Immediate postoperative care comprised administration of dexamethasone (0.1 mg kg^−1^, i.v.) every 24 h, buprenorphine (0.005 mg kg^−1^, i.v.; Buprecare 0. 3 mg mL^−1^ soluble injection; Group Divasa-Farmavic, Barcelona, Spain) every 8 h, head elevation, intravenous fluid therapy (Sterovet; B. Braun Vetcare S.A., Barcelona, Spain) to maintain normotension and regular evaluation of neurological status. Neurological examination was done 24 h after surgery and revealed no worsening of neurological deficits. Neurological signs improved within 5 days and resolved upon hospital discharge (14 days after surgery).

## Discussion

Wider availability of imaging methods (e.g. MRI) to veterinary surgeons and the growing popularity of having goats as domestic pets have resulted in more goats requiring surgery that involves general anaesthesia. Recommendations for patients with intracranial neoplasms have not been determined and management has been extrapolated from studies in head-trauma patients [22]. Many methods for anaesthesia in animals with CNS disease have been reported, but their cerebrovascular effects are unknown.

The main aim of any anaesthetic method used for intracranial surgery is preservation of neuronal function [22]. The latter is dependent upon adequate CBF, which in turn is dependent on the CPP and cerebral vascular resistance (CVR). CPP is defined as the difference between MAP at the level of the base of the brain and ICP. In patients with increased intracranial pressure, it is vital to consider the changes that a decrease in blood pressure may have in CPP [23], so maintaining adequate and stable cardiovascular function is essential.

Tracheal intubation can stimulate coughing and gagging, as well as a reflex sympathetic pressor response, which results in an elevated HR and blood pressure in humans [24], dogs [25] and cats [26]. This reflex may have adverse effects in patients with increased ICP.

Intravenous lidocaine has been used in humans at 1.5–2.5 mg kg^−1^ at least 3 min before the induction of anaesthesia to blunt the haemodynamic and coughing responses to tracheal intubation [27]. In this case, lidocaine administration before intubation to minimise the pressor response to intubation (as has been described in humans) was not done, but non-significant changes were observed in the HR or MAP after intubation. In this case, propofol provided a smooth induction of anaesthesia with no apnoea or myoclonus, as have been reported elsewhere [15, 28].

The position of the head can aid or worsen the control of ICP. Head elevation by 15–30° can improve cerebral venous drainage and limit cerebral venous congestion, thereby reducing ICP without reducing CPP or CBF [29]. Therefore, maintenance of this position is recommended if an increase in ICP is suspected. Occlusion of the jugular vein can impair venous drainage to cause increased ICP. Jugular catheterisation of patients at risk of raised ICP is controversial [29], so we avoided measuring CVP in this case.

In this case, we preferred lactated Ringer’s solution to ensure electrolyte balance despite being a slightly hypotonic fluid and could therefore cause oedema. The MRI did not show evidence of oedema in the surrounding tissue, for this reason we avoided using mannitol preoperatively (as has been recommended). However we used corticosteroids perioperatively to maintain adequate CPP by reducing peri-cyst oedema after surgery, as has been described in the treatment of brain tumours [30].

In the absence of direct monitoring of ICP, it is recommended that a MAP of 80–100 mmHg be maintained to ensure adequate CPP [31]. Maintenance of an adequate depth of anaesthesia to avoid hypotension is very important. In this case, MAP was >80 mmHg most of the time (Fig. 2). Elevated ICP could be a problem until reduction in ICP occurs after durotomy, but no treatment was initiated to increase MAP. Because of the positioning of the animal for surgery, the eye position, jaw tone and palpebral reflexes could not be used to assess the depth of anaesthesia. Furthermore, the usefulness of MAP and HR in the assessment of the depth of anaesthesia is confounded by the effects of anaesthetic agents on cardiovascular function [22]. Despite such difficulties in the assessment of the depth of anaesthesia, the goat started to breathe against the ventilator and received a bolus of propofol only once. In human medicine, electroencephalography is useful for monitoring the adequacy of sedation and anaesthesia during neurosurgery, but an electroencephalograph was not available in our hospital.

The *P*aCO_2_ is an important factor for the control of CVR and CBF. An acute rise in *P*aCO_2_ can cause a decrease in arteriolar pH, which results in decreased CVR and which increases CBF. In contrast, hypocapnia results in intracranial vasoconstriction and decreased cerebral perfusion [29]. IPPV is mandatory in neurosurgical patients to prevent increases in *P*aCO_2_ as well as the associated vasodilatation and increased ICP [32]. Improvements in MAP and CPP may also be achieved by limiting the detrimental effects of IPPV upon venous return and cardiac output [22]. IPPV can increase central venous pressure (CVP) and decrease venous drainage from the head, thereby leading to venous congestion, increased ICP and further reductions in CPP [32]. Court et al. [33] proposed that this effect could be minimised by using neuromuscular blocking agents that increase thoracic compliance and thus decrease the intrathoracic pressure required to ventilate the animal adequately. In this case, we avoided neuromuscular blocking agents but muscle relaxation was sufficient to ensure adequate IPPV with no apparent cardiovascular effects.

Interest in TIVA for neuroanaesthesia has been well documented in the literature on human medicine [10]. Research on TIVA in goats has become increasingly popular over the past few years [13, 21, 34]. However, information on TIVA protocols applicable to goats is very scarce [34].

Pharmacokinetic studies in various species have revealed that propofol has a large volume of distribution, rapid metabolism and rapid clearance if given by repeated doses or continuous intravenous infusion [28, 35, 36]. The rapid onset, short duration of action and rapid recoveries make this drug potentially useful in ruminants, in which these features are particularly desirable [37]. Plasma clearance of propofol in goats is rapid and exceeds the rate clearance in dogs, horses and humans [36]. Propofol has been used as a continuous intravenous infusion to achieve and maintain general anaesthesia in goats [1, 19–21].

The minimum infusion rate of propofol to prevent purposeful movement of the extremities in response to a standardised noxious stimulus in non-premedicated goats is 0.45 mg kg^−1^ min^−1^ [38]. In studies undertaken by Dzikiti et al. [39] and Larenza et al. [20], infusion rates of propofol of 0.2 and 0.3 mg kg^−1^ min^−1^, respectively, were reported. Both studies co-administrated anaesthetic-sparing agents (fentanyl, midazolam [39]; ketamine [20]), to lower the infusion rates of propofol required to maintain anaesthesia. Nevertheless, Carroll et al. [40] reported a higher dose rate (0.52 mg kg^−1^ min^−1^) in pre-medicated (detomidine and butorphanol) goats undergoing orchiectomy and ovariectomy, perhaps because surgical stimulation may exceed the standardised supramaximal stimulus applied in research studies. In our case, the CRI of propofol was 0.2 mg kg^−1^ min^−1^, this can be explain for co-administration of lidocaine, midazolam and fentanyl CRI.

Midazolam produces a rapid onset of short-duration, dose-dependent sedation in goats [14, 15] and decreases the induction doses of propofol required [15]. It is associated with minimal adverse cardiovascular effects if administered as pre-anaesthetic medication [15] and at 0.1–0.9 mg kg^−1^ h^−1^ for an adjunctive role to general anaesthetic drugs in goats [21, 39]. Midazolam produces marked reductions in drug requirements for general anaesthesia in inhalation and intravenous anaesthesia in goats [21, 39, 41]. Sedative effects of midazolam in goats are dose dependent, therefore, in this case, a higher dose of midazolam might have been used to further decrease the dose of propofol required for maintenance.

Lidocaine was infused immediately after intubation throughout surgery to elicit neuroprotective and analgesic effects. Lidocaine may reduce secondary brain injury by preventing sodium influx into ischemic neurons [42]. Evidence suggests that infusion of an antiarrhythmic dose (1.5–2 mg kg^−1^) after the onset of brain ischemia reduces the risk of death of neuronal cells and improves neurologic outcome [43].

The synthetic μ-opioid agonist fentanyl is used for the treatment of moderate-to-severe pain. Fentanyl administered via the intravenous route as CRI after a bolus dose could be a useful analgesic adjunct to general anaesthesia in goats [39]. Fentanyl causes minimal adverse effects on cardiopulmonary function in goats, but has been associated with restlessness, increased vocalisation and exaggerated tail wagging during recovery from anaesthesia [39]. Although we used a low dose of fentanyl, to avoid these possible adverse effects and to ensure a smooth recovery and more compliant animal in the postoperative period, a small dose of acepromazine was administered at the end of the surgery. Despite that acepromazine should cause hypotension and vasodilatation and presumably decrease of CPP, we believe that the effects of an excited recovery would be even more damaging in this goat.

In our case, the recovery time was short and adverse effects such as myoclonus (which has been reported in goats recovering from a single dose of propofol [15]) was not observed, unlike in studies were CRI of propofol was used to maintain anaesthesia [21]. One important feature of a neuroanaesthetic protocol is rapid recovery from anaesthesia to allow prompt neurological assessment [10]. Rapid predictable recovery with minimal excitement regardless of the duration of anaesthesia is required to identify postoperative complications (e.g. haemorrhage, increased ICP) and prevent severe neurological impairment and death [23]. Moreover, rapid recovery from anaesthesia is important in ruminants because they are prone to tympany and regurgitation of ruminal contents, which increases the risk of hypoxemia and aspiration of regurgitated ruminal contents [37].

## Conclusion

We reported on anaesthetic management in a goat undergoing surgical treatment for cerebral coenurosis. This is the first report of CRI of propofol, lidocaine midazolam and fentanyl in goats. TIVA (achieved by co-administration of propofol, lidocaine, midazolam and fentanyl) for the maintenance of anaesthesia in a ventilated oxygen-supplemented goat undergoing craniectomy resulted in satisfactory anaesthesia with minimal negative impact on cardiopulmonary function as well as good recovery. This method could be an acceptable option for anaesthesia during intracranial surgery in goats.

## Abbreviations

CBF: Cerebral blood flow
CMR: Cerebral metabolic rate
CNS: Central nervous system
CPP: Cerebral perfusion pressure
CRI: Continuous rate infusion
CVP: Central venous pressure
CVR: Cerebral vascular resistance
*f*_R_: Respiratory rate
HR: Heart rate
IBP: Invasive blood pressure
ICP: Intracranial pressure
IPPV: Intermittent positive pressure ventilation
MAP: Mean arterial blood pressure
PIP: Peak inspiratory pressure
SpO_2_: Plethysmographic oxygen saturation
TIVA: Total intravenous anaesthesia
PE′CO_2_: Partial pressure of expired carbon dioxide
